# Lnc-C2orf63-4-1 Confers VSMC Homeostasis and Prevents Aortic Dissection Formation via STAT3 Interaction

**DOI:** 10.3389/fcell.2021.792051

**Published:** 2021-12-06

**Authors:** Song Zhang, Shiqi Zhao, Xuejie Han, Yun Zhang, Xuexin Jin, Yue Yuan, Xinbo Zhao, Yingchun Luo, Yun Zhou, Yunlong Gao, Hui Yu, Danghui Sun, Wei Xu, Sen Yan, Yongtai Gong, Yue Li

**Affiliations:** ^1^ Department of Cardiology, The First Affiliated Hospital, Harbin Medical University, Harbin, China; ^2^ Department of Pharmacology (State-Province Key Laboratories of Biomedicine-Pharmaceutics of China, Key Laboratory of Cardiovascular Medicine Research, Ministry of Education), College of Pharmacy, Harbin Medical University, Harbin, China; ^3^ The Cell Transplantation Key Laboratory of National Health Commission, Harbin, China; ^4^ Key Laboratory of Hepatosplenic Surgery, Harbin Medical University, Ministry of Education, Harbin, China; ^5^ Key Laboratory of Cardiac Diseases and Heart Failure, Harbin Medical University, Harbin, China; ^6^ Institute of Metabolic Disease, Heilongjiang Academy of Medical Science, Harbin, China; ^7^ Heilongjiang Key Laboratory for Metabolic Disorder and Cancer Related Cardiovascular Diseases, Harbin, China

**Keywords:** long non-coding RNAs, stat3, vascular remodeling, vascular smooth muscle cells, aortic dissection

## Abstract

Emerging evidence indicates that long non-coding RNAs (lncRNAs) serve as a critical molecular regulator in various cardiovascular diseases. Here, we aimed to identify and functionally characterize lncRNAs as potential mediators in the development of thoracic aortic dissection (TAD). We identified that a novel lncRNA, lnc-C2orf63-4-1, was lowly expressed in aortic samples of TAD patients and angiotensin II (Ang II)-challenged vascular smooth muscle cells (VSMCs), which was correlated with clinically aortic expansion. Besides, overexpression of lnc-C2orf63-4-1 significantly attenuated Ang II-induced apoptosis, phenotypic switching of VSMCs and degradation of extracellular matrix both *in vitro* and *in vivo*. A customized transcription factor array identified that signal transducer and activator of transcription 3 (STAT3) functioned as the main downstream effector. Mechanistically, dual-luciferase report analysis and RNA antisense purification (RAP) assay indicated that lnc-C2orf63-4-1 directly decreased the expression of STAT3, which was depend on the reduced stabilization of STAT3 mRNA. Importantly, up-regulation of STAT3 efficiently reversed the protective role of lnc-C2orf63-4-1 against Ang II-mediated vascular remodeling. Therefore, lnc-C2orf63-4-1 negatively regulated the expression of STAT3 and prevented the development of aortic dissection. Our study revealed that lnc-C2orf63-4-1 played a critical role in vascular homeostasis, and its dysfunction exacerbated Ang II-induced pathological vascular remodeling.

**GRAPHICAL ABSTRACT:**
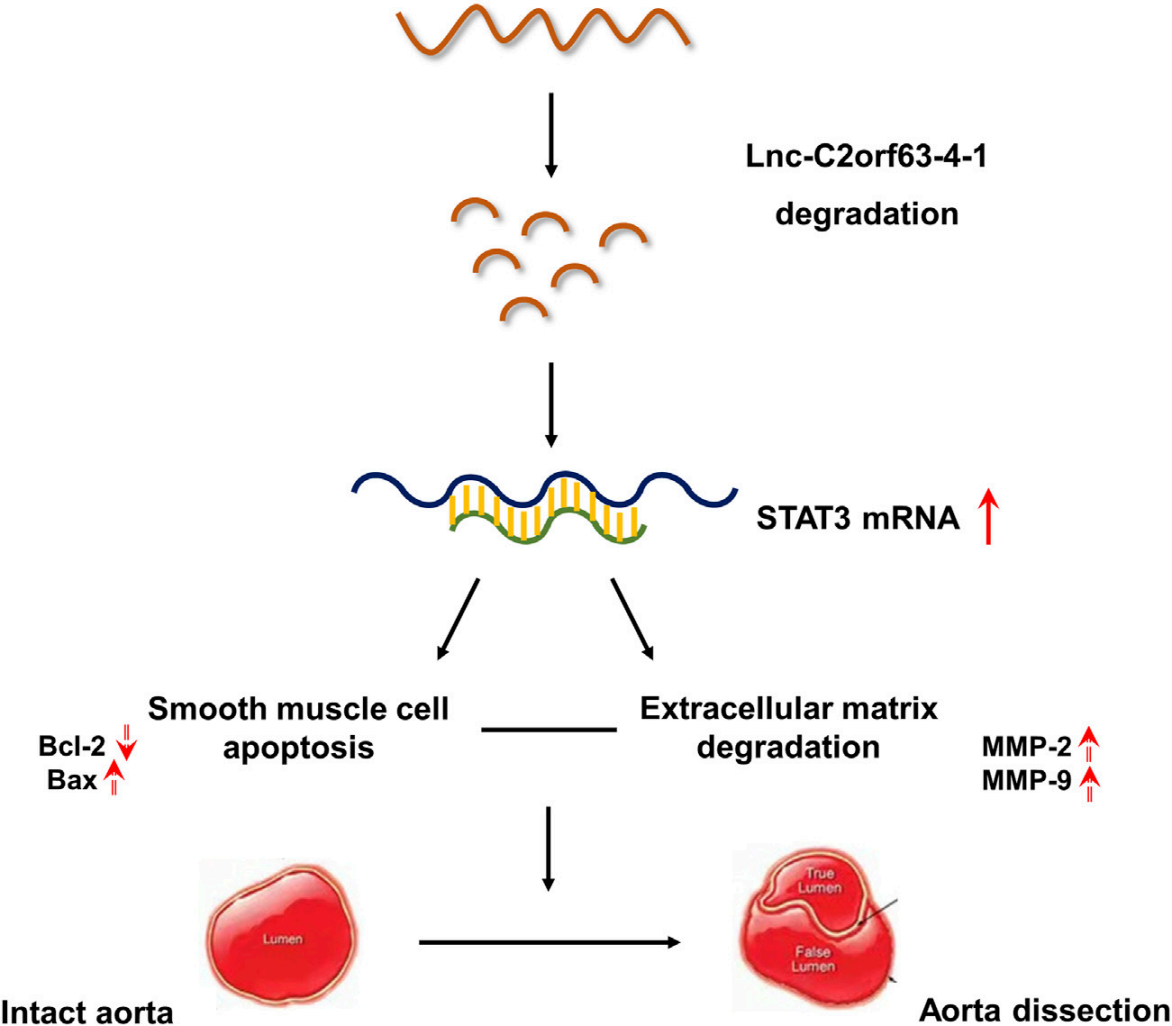
Our studies indicates that lnc-C2orf63-4-1 was a novel regulator of smooth muscle cell survival during the development of aortic aneurysm and dissection (AAD). Under pathological stimulation, decreased levels of lnc-C2orf63-4-1 bind to the promoter region of STAT3 resulting in enhancement of STAT3 mRNA stabilization, which can activate downstream apoptosis signaling (indicated by elevated BAX and decreased BCL2 levels), matrix metalloproteinase family (indicated by elevated MMP-2 and MMP-9 levels) and interstitial fibrosis factors (indicated by elevated type I and type III collagen levels). Taken together, these events trigger smooth muscle cell apoptosis, degradation of ECM, and fibrosis, which induces vascular remodeling and aggravating AAD development.

## Introduction

Thoracic aortic dissection (TAD) is the most serious form of acute aortic syndrome with an incidence of about 15 cases per 100,000/year (Mussa et al., 2016). The characteristic histological features of the aortic wall in TAD patients mainly include medial degeneration, such as damage to the elastic fiber, loss of vascular smooth muscle cells (VSMCs), and disruption and degradation of structural extracellular matrix (ECM) (Nienaber et al., 2016). As the predominant cells in the tunica media of the aorta, VSMCs are essential for the maintenance of the aortic structure and function. Increasing evidence shows that enhanced apoptosis of VSMCs leads to the development of many aortic diseases due to vascular remodeling (Rzucidlo et al., 2007). Therefore, it is necessary to identify and characterize molecules involved in the regulation of VSMC apoptosis to prevent pathological vascular remodeling. Despite advanced surgical techniques and new therapeutic strategies, the mortality rate for TAD remains between 10 and 35%, even at experienced medical centers (Go et al., 2013). It is essential to obtain a better understanding of the cellular mechanisms and regulatory networks driving TAD development and progression to identify novel therapeutic targets.

The advancement in large-scale whole-genome sequencing technologies suggests that less than 2% of the human genome encodes for proteins, whereas much of the remaining genome is transcribed into non-coding RNAs (ENCODE Project Consortium, 2012). Long non-coding RNAs (lncRNAs) refer to a class of RNA transcripts longer than 200 nucleotides with limited protein-coding potential. LncRNAs regulate gene expression, including chromatin remodeling, mRNA transcription and processing, and post-transcriptional pathways, which function as powerful mediators in all aspects of molecular regulation under physiological and pathological conditions (Duggirala et al., 2015). Recently, studies have shown that various lncRNAs, such as H19 (Ren et al., 2020), CDKN2B-AS1 (Zhao et al., 2020), XIST (Zhang et al., 2020), PTENP1 (Lai et al., 2019), and AK056155 (Yu et al., 2015), are involved in the pathological damage of the aorta by regulating the VSMC apoptosis and ECM degradation, playing a critical role in the pathophysiologic processes of vascular remodeling. However, the contribution of lncRNAs to the development of vascular remodeling in TAD has not been experimentally addressed. In the present study, we aimed to evaluate if and how lncRNAs were involved in such a process.

In the present study, we utilized next-generation high-throughput sequencing (HTS) for TAD patients and control subjects (healthy organ donors) and found that the expression of lnc-C2orf63-4-1 was reduced during the development of aortic dissection. Moreover, we showed that overexpression of lnc-C2orf63-4-1 significantly prevented the incidence and severity of aortic dissection in angiotensin II (Ang II)-infused mice, potentially via decreasing VSMC apoptosis, matrix metalloproteinase (MMP) activity, and accumulation of collagen fibers. Specifically, we identified that lnc-C2orf63-4-1 affected aortic dissection through mediating the function of signal transducer and activator of transcription 3 (STAT3), which exerted pro-apoptotic effects in VSMCs, leading to subsequent vascular remodeling.

## Materials and Methods

### Patient Selection and Collection of Aortic Tissues

Study protocols were approved by the ethical committees of the First Affiliated Hospital of Harbin Medical University (No. HMUIRB20170034) and complied with the declaration of Helsinki. Written informed consents were obtained from all participants.

The tissue samples of ascending aorta were collected from Stanford type A aortic dissection patients (*n* = 24), who were identified without Marfan syndrome, Loeys-Dietz Syndrome, familial aortic dissection, and bicuspid aortic valve. A reference control group (*n* = 13) was set up from patients who had coronary artery bypass grafting (CABG; *n* = 10) and heart transplant donors (*n* = 3) without hereditary cardiovascular disease, aortic dissection, previous history of inflammatory aortic disease, or known connective tissue disorder.

The specimens were immediately sliced into small pieces, soaked in RNAlater solution (ThermoFisher Scientific, United States), and preserved at −80°C until further assays. The gene expression analysis was performed using lncRNA in the aortic wall from Stanford type A aortic dissection patients (*n* = 3) and heart transplant donors (*n* = 3). The other aortic wall samples from control and TAD groups were prepared for quantitative real-time polymerase chain reaction (qRT-PCR), Western blotting analysis, and histological analysis. Supplementary Table S1 lists the characteristics of patients who provided tissue samples.

### RNA Extraction and Quality Control

Total RNA was extracted using RNAiso Plus Total RNA extraction reagent (Cat# 9109, TAKARA) following the manufacturer’s instructions, the RNA integrity was examined using an RNA integrity number (RIN) by an Agilent Bioanalyzer 2100 (Agilent Technologies, Santa Clara, CA, United States), and samples with an initial RIN >6.5 were used for the gene expression profiling. Qualified total RNA was further purified by RNAClean XP Kit (Cat# A63987, Beckman Coulter, Inc., Kraemer Boulevard Brea, CA, United States) and RNase-Free DNase Set (Cat#79254, QIAGEN, GmBH, Germany).

### RNA-Seq and Data Assay

Aortic wall tissues were processed to synthesize double-stranded complementary DNA (cDNA), and the cDNA was labeled and hybridized to lncRNA by using an Illumina HiSeq 2500 (Illumina, Santiago, CA, United States) at Shanghai Biotechnology Co., Ltd. (Shanghai, China). The expression levels of whole samples were presented as Fragments Per Kilobase of exon model per Million mapped fragments (FPKM) values, which is the recommended and most commonly used method to estimate the level of gene expression (Mortazavi et al., 2008). The differentially expressed genes were selected with the expression threshold of fold change >2.0 or <0.5 and Benjamini-Hochberg corrected *p* values of <0.05.

### Validation of Gene Expression by qRT-PCR

qRT-PCR was performed to validate the HTS results. Bioinformation website (http://www.noncode.org/) showed that nine lncRNAs were conservative with mice, and we further randomly selected seven among these differentially expressed lncRNA transcripts for validation. Total RNA was extracted from the control (*n* = 13) and TAD (*n* = 24) aortic tissues using TRIzol reagent (Invitrogen Life Technologies, Carlsbad, CA, United States) and then reversely transcribed to cDNA. qRT-PCR was conducted using a standard SYBR Green PCR kit (Toyobo, Japan) on an ABI 7500 real-time PCR System (Applied Biosystems, United States). β-actin was selected as the housekeeping gene, and all experiments were performed in triplicate. The relative expressions of the target genes were calculated by the 2^−ΔΔCt^ method. The operations were carried out as previously described (Sun et al., 2015). The primers of related genes used in the study were listed in Supplementary Table S2.

### Cell Cultures and *in vitro* Experiments

Mouse VSMCs were purchased from ATCC (CRL-2797, Manassas, VA, United States) and maintained in DMEM (Gibco Laboratories, NY, US) supplemented with 10% fetal bovine serum, 1% penicillin, and 1% streptomycin (Gibco-BRL, Rockville, MD, United States) at 37°C in a humidified atmosphere containing 5% CO_2_. To specifically overexpress or inhibit lnc-C2orf63-4-1, overexpress or inhibit STAT3, the lentivirus vector was purchased from Genechem Inc. (Shanghai, China) (Yuan et al., 2018). Cells were transfected with 2 μl (1 × 10^8^ TU/ml) of overexpression lentivirus (OE), inhibition of expression lentivirus carrying shRNA (sh), or empty lentivirus (NC) after 24 h of plating. The blank group was transfected with the same volume of culture medium. At 72 h after the lentivirus were transfected into VSMCs, the Ang II (Sigma, St. Louis, MO, United States) at a concentration of 1,000 nM (Zhao et al., 2017) was added to cells, followed by incubation for another 24 h.

### RNA-FISH Assay

RNA fluorescence *in situ* hybridization (RNA-FISH) Cy3-labeled lnc-C2orf63-4-1 was purchased from RiboBio (Guangzhou, China). Frozen tissues were sectioned by Cryotome E (Thermo Fisher Scientific, Waltham, MA, United States). The final signal was developed with diaminobenzidine (Dako, Copenhagen, Denmark), and the nuclei were counterstained in hematoxylin for 3 min. The sequence of lnc-C2orf63-4-1 probe used was as follows: 5′-TCG​TCT​AAA​CAA​ACA​TTT​CAT​TTC​AGA​AAA​TCT​GCA​TCA​ATC​TAC​ACG​GAC-3′.

### RAP Assay

RNA antisense purification (RAP) was performed based on a previously published method (McHugh et al., 2015). The lysates were centrifuged at 3,300 ×g for 7 min at 4°C, and the pellets containing nuclei were resuspended in 1 ml of GuSCN Hybridization Buffer (20 mM Tris-HCl pH 7.5, 7 mM EDTA, 3 mM EGTA (Sigma, cat# E3889-10G), 150 mM LiCl (Sigma, cat# 62476-100G-F), 1% NP-40 (Sigma, cat# I8896-100ML), 0.2% N-lauroylsarcosine (Sigma, cat# L7414-10ML), 0.1% sodium deoxycholate (Sigma, cat# D6750-25G), 3 M guanidine thiocyanate (Sigma, cat# G9277-100G), and 2.5 mM TCEP). Probes were subsequently captured by incubation with streptavidin-coated magnetic beads for 30 min at 37°C with constant mixing, followed by magnetic separation. After washing, the beads were magnetically separated and washed with RNase Helution buffer (50 mM Tris-HCl, pH 7.5, 75 mM NaCl, 3 mM MgCl_2_, 0.125% N-lauroylsarcosine, 0.025% sodium deoxycholate, and 2.5 mM TCEP). Eluted RNA complexes were used in qRT-PCR to quantify the RNA yield and enrichment.

### Luciferase Reporter Assay

STAT3-WT and STAT3-Mut were cloned into the luciferase vector V80-PmirGLO (HeChuang Bio Co., Ltd., Guangzhou, China). For luciferase reporter assays, the lnc-C2orf63-4-1 was co-transfected into mouse aortic VSMCs with the luciferase constructs described above using Lipofectamine 2000 (Invitrogen, Thermo Fisher Scientific). The luciferase activity at 24 h post-treatment was measured by the Dual-Luciferase Reporter Assay System (Promega, Madison, WI, United States) as previously described (Eken et al., 2017).

### Cell Apoptosis Assay

Apoptosis was determined by flow cytometry analysis using the Annexin V-FITC/PI Apoptosis Kit (Becton Dickinson, Franklin Lakes, NJ, United States). Cells were seeded in 6-well plates (5 × 10^5^ cells/well), digested with trypsin (Gibco trypsin-EDTA; Thermo Fisher Scientific), washed with phosphate-buffered saline (PBS) three times, suspended in 500 μl of binding buffer, and then incubated with 5 μl of fluorescein isothiocyanate (FITC)-conjugated Annexin V and 3 μl of propidium iodide (PI) for 15 min at room temperature in the dark. After incubation, the samples were tested using a flow cytometer (Moflo XDP, United States). Early apoptotic chondrocytes were defined as annexin V-positive and PI-negative cells, and late apoptotic chondrocytes were defined as annexin V-positive and PI-positive cells. The proportion of apoptotic cells was calculated using the FASC Calibur MT flow cytometer (BD Bioscience, NJ, United States).

### Western Blotting Analysis

Different groups of aortic tissues and VSMCs were collected. Subsequently, tissues or cells were lysed on ice with a radioimmunoprecipitation assay buffer containing protease inhibitors. The protein concentration was determined with an Enhanced BCA Protein Assay Kit. The lysates were boiled at 100°C for 10 min. Equal amounts of proteins were subjected to sodium dodecyl sulfate-polyacrylamide gel electrophoresis and then transferred onto PVDF membranes. The membranes were blocked with 5% skim milk at room temperature for 1 h and incubated with a specific primary antibody at 4°C overnight. Then the membranes were incubated with the secondary antibody at room temperature for 1 h. Finally, immunoreactive bands were visualized using the enhanced chemiluminescence (ECL) chromogenic substrate at room temperature for 2 min, and the band density was quantified using Image Lab software.

### Histology Assay

Histological staining was performed by using a standardized protocol as previously described. Aortas were isolated and fixed in 4% formalin overnight at 4°C. Paraffin cross-sections (4 μm) from organs were stained with hematoxylin and eosin, elastic van Gieson (EVG) staining, and Masson staining. The interstitial fibrotic areas were calculated using Image-Pro Plus software (version 4.0; Media Cybernetics LP). Collagen volume fraction was calculated as collagen area/total area ×100%.

### Ang II-Infused AAD Model

All animal experiments were reviewed and approved by the Animal Care and Use Review Committee of Harbin Medical University (Animal Experimental Ethical Inspection Protocol No. HMUIRB20170034). The study conformed to the *Guide for the Care and Use of Laboratory Animals* published by the US National Institutes of Health. Mice were anesthetized using 2% isoflurane (Vet One) and laid supine on a heated plastic pillow (37°C), the osmotic minipumps were implanted after used microscopic scissors to cut the back skin. The osmotic minipumps (model 2004, Alzet) containing either Ang II (1 μmol/kg/min, Sigma-Aldrich) to construct aortic aneurysm and dissection (AAD) model or saline were implanted in 22∼26 week-old ApoE^−/−^ male mice (control group). Only male mice were used because of the male predominance of human AAD and the potential influence of female sex hormones on AAD models (Golledge et al., 2006). Adeno-associated virus (AAV) has been identified as the most promising gene therapy vehicle due to its advantages, including efficient infection, non-pathogenicity, broad tissue transduction, and long-term gene expression (Zincarelli et al., 2008). Among various serotypes, AAV9 is the most efficient vector for vascular transduction (Koblan et al., 2021). At 3 days before osmotic mini-pumps were implanted, mice were intravenously administered with a single dose with 10^12^ genome copies of AAV9 encoding lnc-C2orf63-4-1 sequence in murine (http://www.noncode.org/) via the tail vein for lnc-C2orf63-4-1 overexpression (AAD + OElnc-V group) (*n* = 10) or control vectors (AAD + NC-V group) (*n* = 10) in 0.1 ml of PBS, or the same volume of PBS (AAD group) (*n* = 10). The pAAV9-CMV-MCS-lnc-C2orf63-4-1 and pAAV9-CMV-MCS-3FLAG-WPRE vectors were generated and packaged by OBiO Technology (Shanghai, China) Co., Ltd. Following final transthoracic echocardiography, mice were sacrificed by intravenous injection of a lethal dose of pentobarbital sodium (100 mg/kg), and aortic sections were removed for further experiments. The aortic dissection in AngII treated mice were defined by histopathological staining. When rupture and thrombosis of the media aorta were observed under light microscope during the autopsy, which confirmed that the mice were occurred aortic dissection.

### Aortic Diameter Measurements by Ultrasound Imaging

At 28 days after Ang II induction, the aortic arch diameter was detected by B-mode US imaging. Mice were anesthetized using 2% isoflurane (Vet One) and laid supine on a heated plastic pillow (37°C). The two-dimensional B-mode US imaging was performed after setting up a real-time micro-visualization scan head (RMV 704) with a central frequency of 40 MHz, a frame rate of 30 Hz, a focal length of 6 mm, and a 20 × 20 mm field of view (VisualSonics). All images were acquired for multiple cardiac cycles and digitally stored in the hard drive for offline analysis. All aortic diameters were measured in anterior-posterior direction during the diastolic phase. US image analysis was performed using the accompanying Vevo770 software (VisualSonics). Measurements were repeated on two separate occasions using a random selection of each dataset and operator blinding to prevent recall bias. For these parameters, results were validated by a comparative analysis of one independent observer blinded to the treatment groups.

### Statistical Analysis

Data were presented as means ± SEM, unless stated differently. Groups were compared using Student’s t-test. Normality was tested to ensure that parametric testing was appropriate. When comparing multiple groups, data were analyzed using analysis-of-variance (ANOVA) with Bonferroni’s post-test. The association of the two variables was evaluated using a two-tailed Pearson’s correlation analysis. A value of *p* < 0.05 was considered statistically significant.

## Results

### Differential Expression of LncRNAs in Human TAD Tissue

**FIGURE 1:**
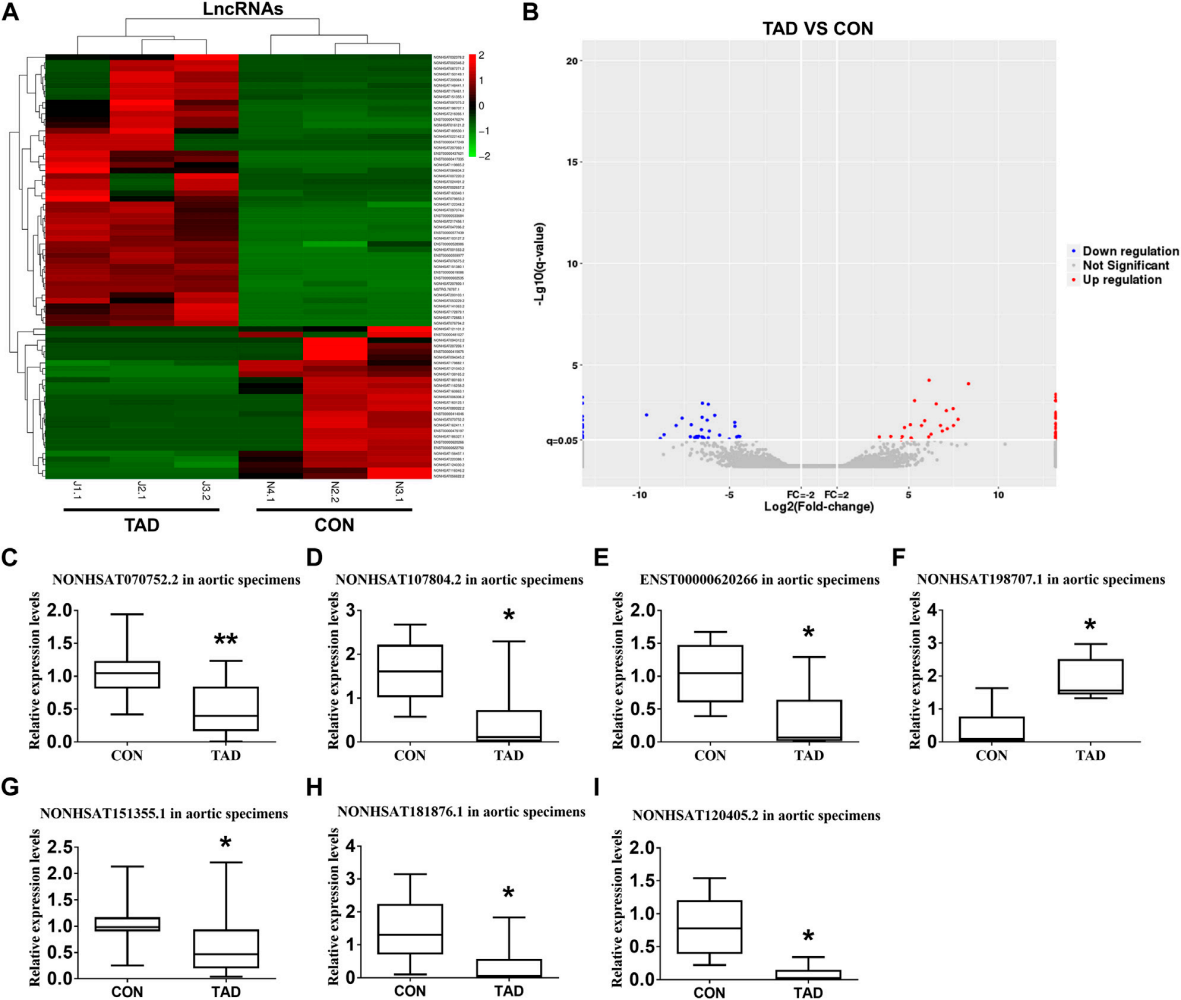
Differentially expressed lncRNAs between TAD patients and healthy controls. **(A)** The hierarchically clustered heatmap illustrating differentially expressed lncRNAs between TAD patients and healthy controls. Up-regulated lncRNAs are denoted in red and down-regulated in green (*n* = 3 per/group). **(B)** The volcano plot illustrates the differentially expressed lncRNAs between the TAD patients and healthy controls, with red dots representing up-regulated genes and blue dots representing down-regulated genes (log2fold change ≥2; q ≤ 0.05) (*n* = 3 per/group). **(C–I)** qRT-PCR was used to compare the relative expression levels of seven aberrantly expressed lncRNAs (NONHSAT107804.2, ENST00000620266, NONHSAT120405.2, NONHSAT181876.1, NONHSAT070752.2, NONHSAT151355.1, and NONHSAT198707.1) (*n* = 15 in the control group, n = 24 in the TAD group). The β-actin was selected as a housekeeping gene. Con represents normal aortic tissue from healthy donors; TAD represents aortic dissection tissue from TAD patients. Data **(C–I)** were expressed as mean ± SEM and compared by Student’s t-test. *p < 0.05.

We comprehensively analyzed the expression profile of lncRNAs in TAD aortic wall tissues by using the HTS analysis and compared it with that in normal aortic wall tissues from healthy donors. A total of 53 lncRNAs (24 up-regulated and 29 down-regulated) were significantly aberrantly expressed in TAD tissues compared with the normal tissues (fold-change ≥ 2, *p* < 0.05). Supplementary Table S3 shows the top 10 ranked up-regulated and down-regulated differentially expressed lncRNAs. The differentially expressed lncRNAs between TAD and healthy donors were illustrated by a hierarchically clustered heat map and volcano plots (Figures 1A,B). To validate the gene expression profiles obtained via lncRNA HTS, we assessed and compared the expressions of seven target genes in aortic specimens obtained from the 24 TAD patients and 15 normal aortic individuals (nine patients with CABG and six healthy donors). All seven genes were significantly up- or down-regulated in the TAD group. Notably, lnc-C2orf63-4-1 (NONCODE: NONHSAT070752.2) was significantly differentially expressed compared with the remaining lncRNAs (Figures 1C–I). Therefore, we focused on this uncharacterized lnc-C2orf63-4-1.

### Lnc-C2orf63-4-1 is Down-Regulated in TAD and Associated With Aortic Expansion

**FIGURE 2:**
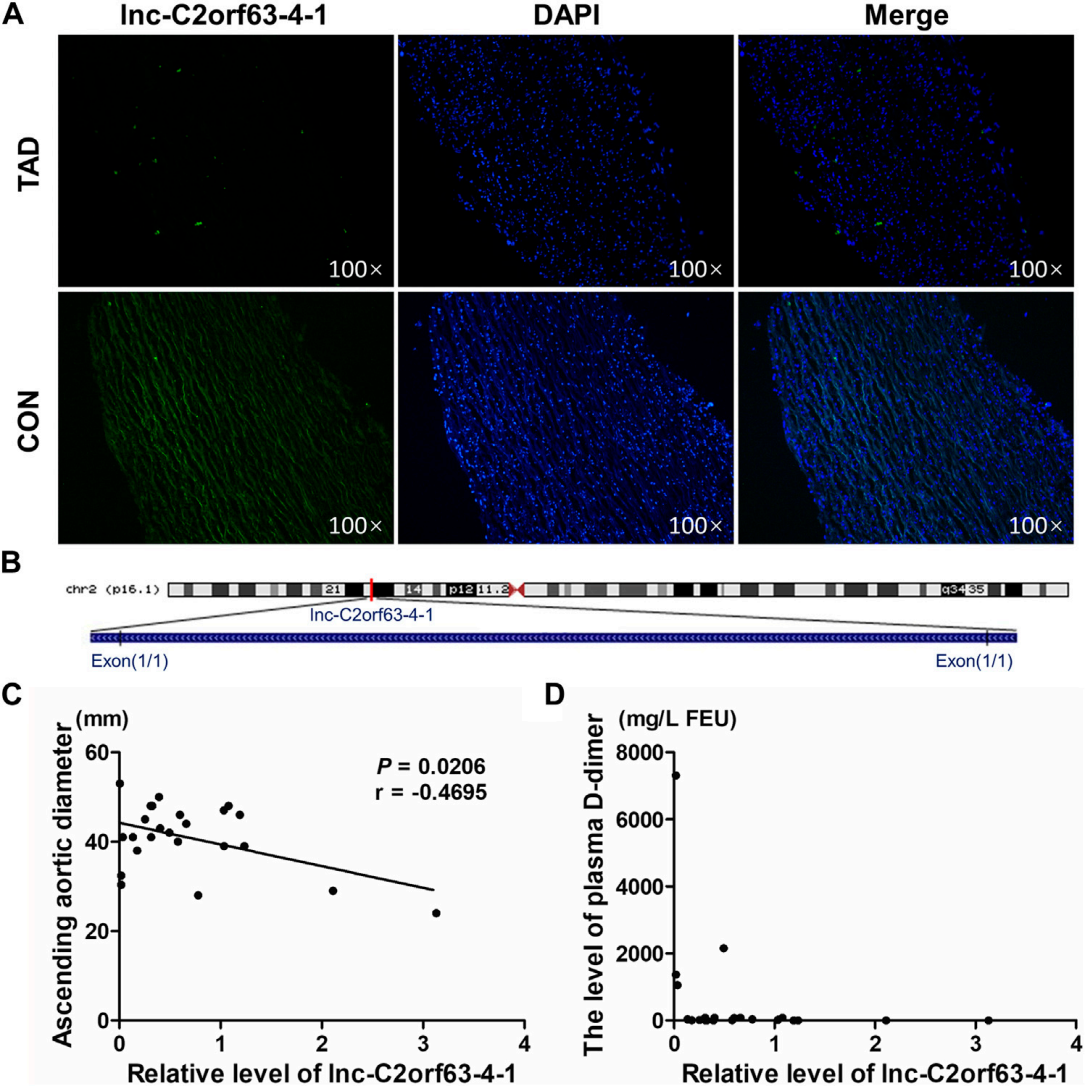
The reduced expression of lnc-C2orf63-4-1 is associated with the aortic arch width in clinical TAD patients. **(A)** RNA-FISH for lnc-C2orf63-4-1 in isolated aorta tissues in TAD patients and healthy controls, clearly suggesting that lnc-C2orf63-4-1 was down-regulated in TAD (*n* = 3/per group). **(B)** Schematic annotation of lnc-C2orf63-4-1 genomic locus on chromosome 2q54.972.558-54.972.776 in humans. The length is 218nt. **(C)** The correlations between the lnc-C2orf63-4-1 expression and ascending aorta diameter of TAD were shown by Pearson’s correlation coefficient (*n* = 15 in the control group, *n* = 24 in the TAD group). **(D)** The correlations between the lnc-C2orf63-4-1 expression and serum D-dimer levels of TAD were shown by Pearson’s correlation coefficient (*n* = 15 in the control group, *n* = 24 in the TAD group).

We next isolated human aortic tissue from TAD and healthy donors and confirmed by RNA-FISH that lnc-C2orf63-4-1 was predominantly localized in the cell cytoplasm but not in the nucleus (Figure 2A). Lnc-C2orf63-4-1 was located on chromosome 2 in humans, which was composed of one exon with a full length of 218 nt (Figure 2B). The non-coding nature of lnc-C2orf63-4-1 was confirmed by coding-potential analysis (Supplementary Figure S1). In addition, we statistically evaluated the correlation between clinical severity factors of TAD, such as aortic arch width and plasma D-dimer concentration, and the expression of lnc-C2orf63-4-1. The results showed negative correlations between aortic arch width and lnc-C2orf63-4-1, and there were no correlations between plasma D-dimer concentration and lnc-C2orf63-4-1 (Figures 2C,D). These data suggested that lnc-C2orf63-4-1 may participated in promoting expansion and rupture of the aorta.

### Overexpression of Lnc-C2orf63-4-1 Inhibits Ang II-Induced VSMC Apoptosis *in vitro*

**FIGURE 3:**
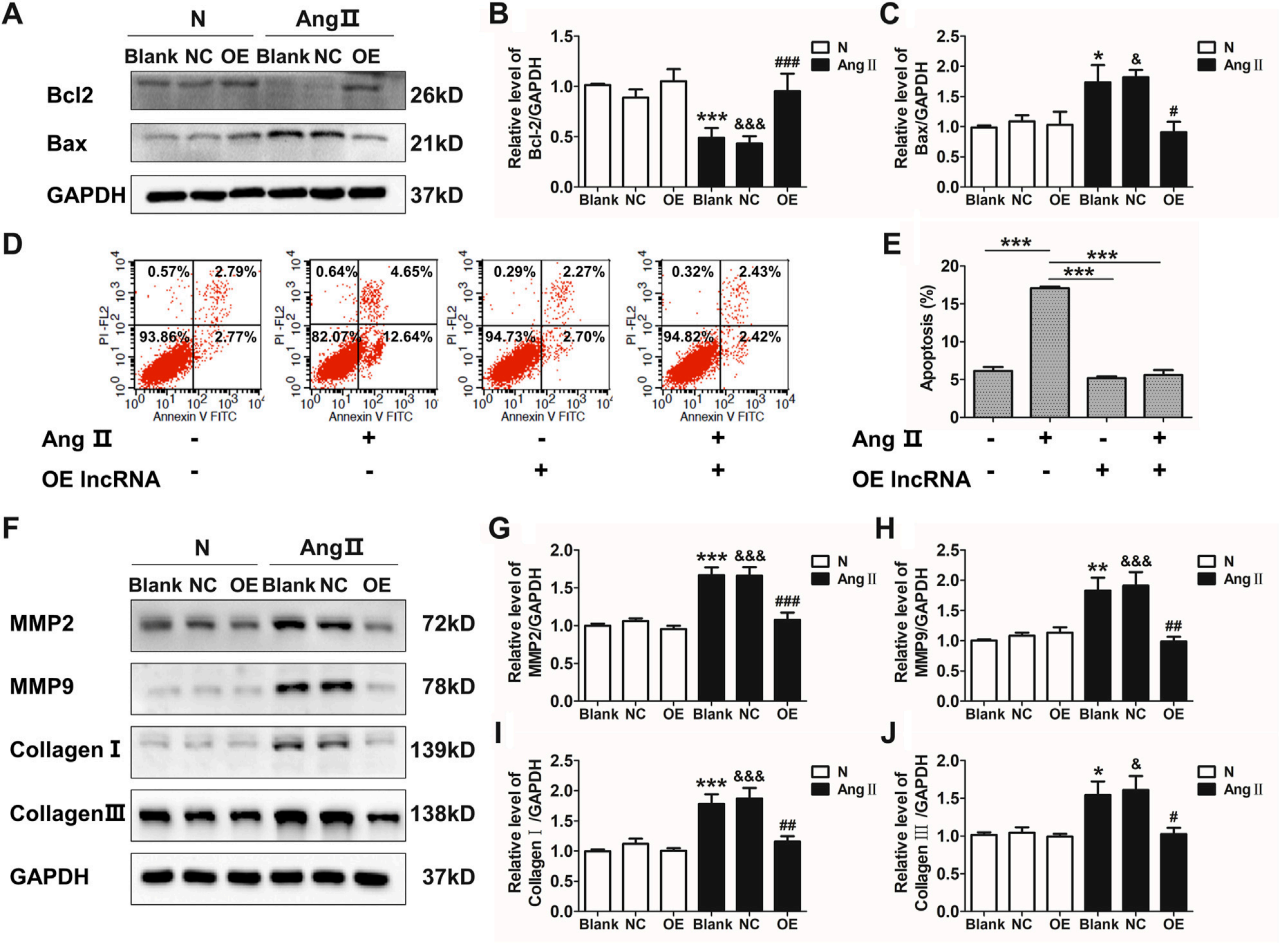
Overexpression of lnc-C2orf63-4-1 attenuates the AngII-induced apoptosis, fibrosis, and MMP-2/9 activity in VSMCs. **(A–C)** Lnc-C2orf63-4-1 reduced the expression of Bax and increased the expression of Bcl-2 in mouse VSMCs following AngII treatment (*n* = 6/per group). **(D,E)** Apoptosis analysis examined by flow cytometry using Annexin V/PI assay (*n* = 4/per group). **(F–J)** Representative WBs and quantification results of MMP-2, MMP-9, type I collagen and type III collagen in the mouse VSMCs treated with or without lnc-C2orf63-4-1 after AngII treatment (*n* = 6/per group). GAPDH was used for normalization. Data were all compared by analysis-of-variance (ANOVA) with Bonferroni’s post-test. *p < 0.05, **p < 0.01, ***p < 0.001 vs. blank group, ^#^ *p* < 0.05, ^##^ *p* < 0.01, ^###^ *p* < 0.001 vs. AngII group, ^&^ *p* < 0.05, ^&&&^ *p* < 0.001 vs. AngII + OE-lnc-C2orf63-4-1 group. Data were all expressed as mean ± SEM.

Ang II has been well documented to induce vascular remodeling. qRT-PCR confirmed that Ang II induced the down-regulation of lnc-C2orf63-4-1 in VSMCs in a time-dependent manner (Supplementary Figure S2A). The expression of lnc-C2orf63-4-1 was significantly elevated after transfection with lentiviral vectors carrying lnc-C2orf63-4-1 sequence in VSMCs (Supplementary Figure S2B). To further explore the function of lnc-C2orf63-4-1 in the vascular remodeling, we investigated its role in the VSMCs under pathological conditions by Ang II. After exposure to Ang II, the expression of pro-apoptotic protein Bcl-2 associated X (Bax) in VSMCs was sharply increased, while anti-apoptotic mediator B-cell CCL/lymphoma (Bcl-2) was significantly down-regulated (Figures 3A–C). In addition, flow cytometry was used to measure the number of apoptotic cells (AV-positive). The results revealed that the proportion of apoptotic cells was increased by Ang II exposure in VSMCs, while it was restored by lnc-C2orf63-4-1 (Figures 3D,E).

One of the characteristic features of AAD is the disruption and degradation of structural ECM proteins by MMPs, particularly MMP-2 and MMP-9 (Longo et al., 2002). Western blotting analysis demonstrated that lnc-C2orf63-4-1 significantly reduced the expressions of MMP-2 and MMP-9 induced by Ang II treatment (Figures 3F–H). In addition, the expressions of collagen I and collagen III, which have been repeatedly demonstrated to contribute to the pathogenesis of vascular remodeling, were also significantly increased in VSMCs by Ang II, while lnc-C2orf63-4-1 inhibited such up-regulation (Figures 3F–J). Therefore, these data suggested that lnc-C2orf63-4-1 participated in Ang II-induced vascular remodeling through influencing VSMC homeostasis.

### Overexpression of Targeting Lnc-C2orf63-4-1 Limits Ang II-Induced Aortic Aneurysm and Dissection Growth in ApoE^−/−^ Mice

**FIGURE 4:**
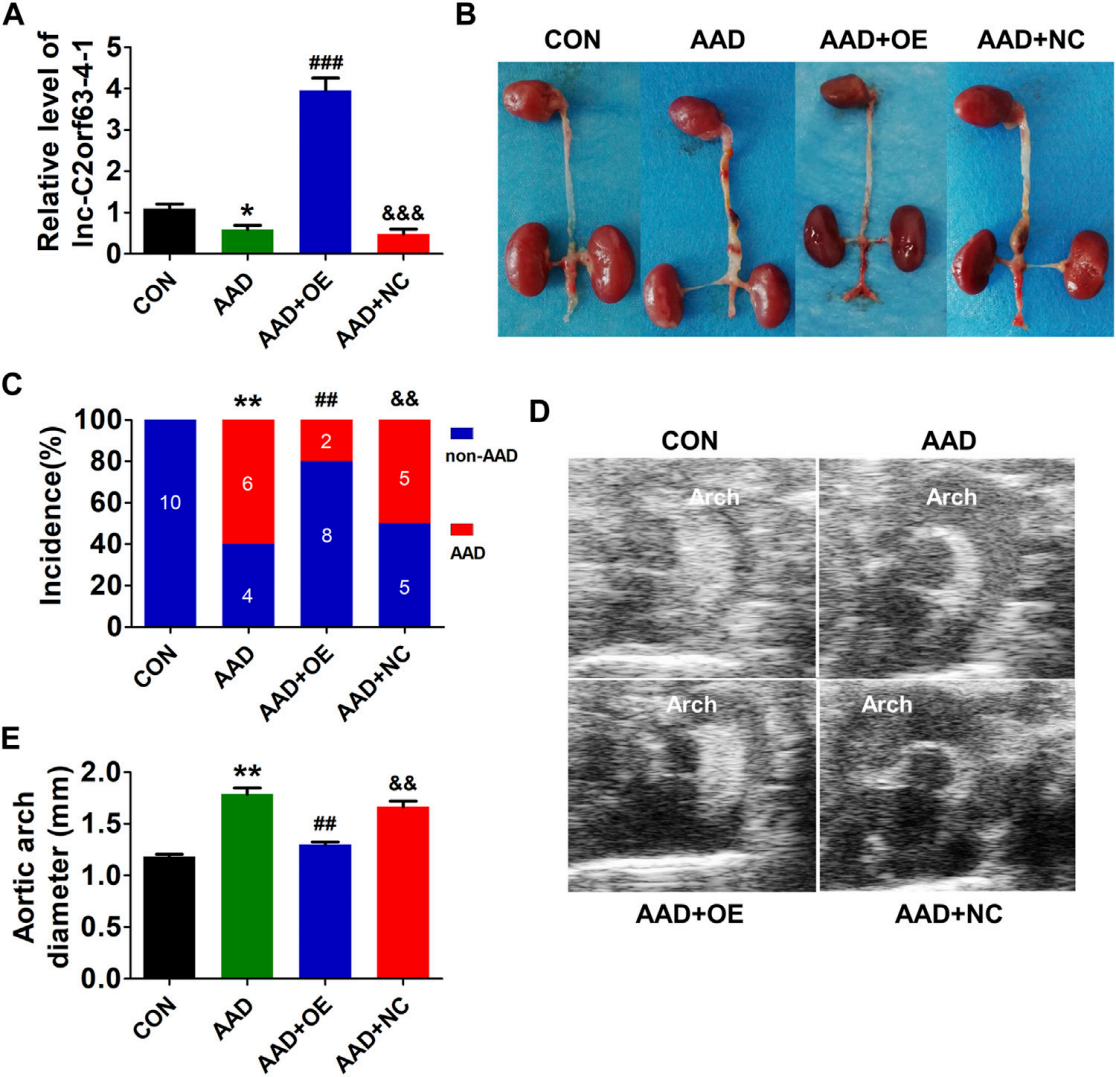
*In vivo* targeting of lnc-C2orf63-4-1 reduces AngII-induced AAD in ApoE^−/−^ mice. **(A)** The expression of lnc-C2orf63-4-1 in aortic tissues in all groups was detected by qRT-PCR (*n* = 10/per group). U6 was used for normalization. **(B)** Representative photographs of AAD in male ApoE^−/−^ mice. AngII (1,000 ng/kg/per min) was chronically infused for 28 days, and saline was used as the control. **(C)** Incidence of AAD in all groups (*n* = 10/per group). **(D,E)** Representative pictures from ultrasonography **(D)** and quantification of the maximal aortic arch **(E)** of all groups at the end of 28 days saline or AngII infusion (*n* = 4–10/per group). Data of **(A)** and **(E)** were compared by analysis-of-variance (ANOVA) with Bonferroni’s post-test. Incidence of aortic dissection **(C)** was presented as numbers and compared by using Fisher’s exact test. *p < 0.05, **p < 0.01 vs. control group, ^##^ *p* < 0.01, ^###^ *p* < 0.001 vs. AAD group, ^&&^ *p* < 0.01, ^&&&^ *p* < 0.001 vs. AAD + OE group. Data were all expressed as mean ± SEM. Con represents saline treatment ApoE^−/−^ mice group; AAD represents aortic dissection and aneurysm model group by ApoE^−/−^ mice treatment with AngII; OE, overexpression of lnc-C2orf63-4-1 by the recombinant adeno-associated virus (serotype 9; AAV9) vector carrying the full-length of lnc-C2orf63-4-1 gene; NC, negative control flag gene engineered into the AAV9 vector. The viral constructs were intravenously injected into mice.

Chronic infusion of Ang II recapitulates many aspects of aortic dissection and is widely used to study AAD (Daugherty et al., 2000; Xu et al., 2019). Here, ApoE^−/−^ mice were injected with AAV9-lnc-C2orf63-4-1 or negative control (NC) construct NC-V (the NC RNA engineered into the AAV9 vector), and they were infused with Ang II for 4 weeks to develop AAD. The efficiency of AAV-lnc-C2orf63-4-1 to rise endogenous lnc-C2orf63-4-1 in AAD mice was also confirmed by qRT-PCR, showing that the transcript level of lnc-C2orf63-4-1 in the aorta was significantly repressed in the aortic dissection group, while it was increased by AAV9-lnc-C2orf63-4-1 treatment (AAD + OElnc-V) (Figure 4A). In the aortic dissection groups, six in 10 Ang II-treated mice displayed expanded aortic arch to the extent of AAD, whereas none of the control mice displayed AAD formation (Figure 4B). More importantly, the incidence of AAD was substantially reduced in the AAD + OElnc-V group (Figure 4C). In addition, 4 weeks of Ang II treatment resulted in a vascular expansion in injured aortas in the AAD group, and the maximal aortic arch diameters were also significantly reduced in the AAD + OElnc-V group (Figures 4D,E **)**, suggesting that overexpression of lnc-C2orf63-4-1 in VSMCs partly attenuated Ang II-induced AAD.

### Up-Regulation of Lnc-C2orf63-4-1 in VSMCs Restores the Aortic Elastic Fiber Degradation and Collagen Disposition in AAD Mice

**FIGURE 5:**
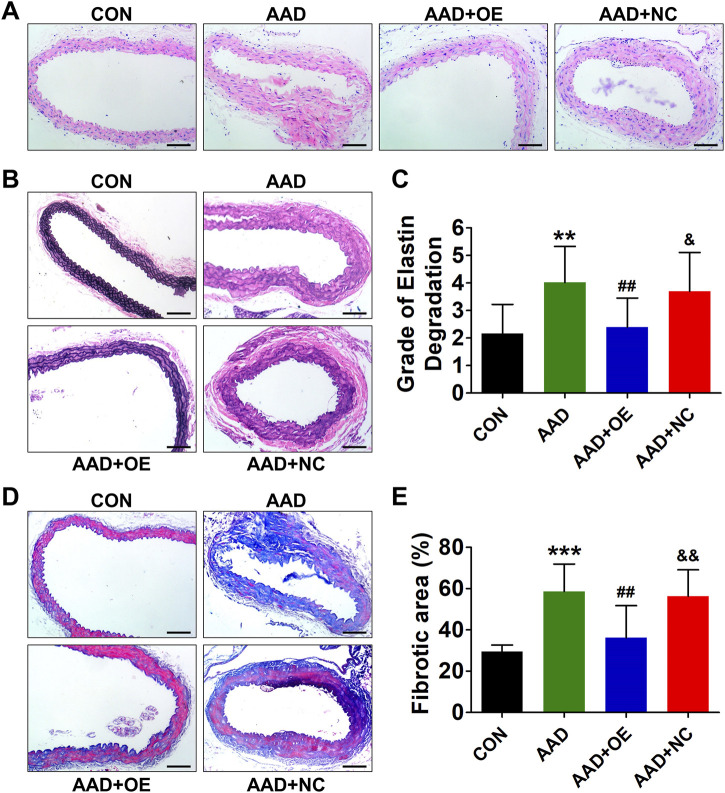
Overexpression of lnc-C2orf63-4-1 prevents AngII-induced aortic elastin fiber degradation and collagen deposition in aortas of ApoE^−/−^ mice. **(A)** Representative hematoxylin and eosin staining of the aortas in all groups (*n* = 4–6/per group). **(B)** Representative EVG staining of aortic elastin in all groups (*n* = 4–6/per group). **(C)** Quantification of aortic elastin degradation in **(B)**. **(D,E)** Representative Masson’s trichrome staining images and the fibrotic area percent of aorta tissues in all groups are shown (*n* = 4/per group). Scale bars = 50 μm. Data of **(C)** and **(E)** were compared by analysis-of-variance (ANOVA) with Bonferroni’s post-test. **p < 0.01, ***p < 0.001 vs. control group, ^##^ *p* < 0.01 vs. AAD group, ^&^ *p* < 0.05, ^&&^ *p* < 0.01 vs. AAD + OE group. Data were all expressed as mean ± SEM.

Histopathological analysis revealed typical aortic expansion and thrombosis in ApoE^−/−^ mice receiving chronic treatment of Ang II, while AAV9-lnc-C2orf63-4-1 remarkedly reduced the thickness of tunica media (Figure 5A). EVG staining of aortic sections of the AAD group showed more severe media degeneration, including elastic fiber fragmentation, stiffness, and disorganization, compared with the control group, which were attenuated by overexpression of lnc-C2orf63-4-1 (Figures 5B,C). Moreover, Masson staining showed that the collagen content in the aorta was also significantly reduced in the AAD + OElnc-V group compared with AAD group. (Figures 5D,E). These findings suggested that activation of lnc-C2orf63-4-1 could protect VSMCs against apoptosis and switching to synthetic phenotype, which leads to the development of AAD *in vivo*.

**FIGURE 6:**
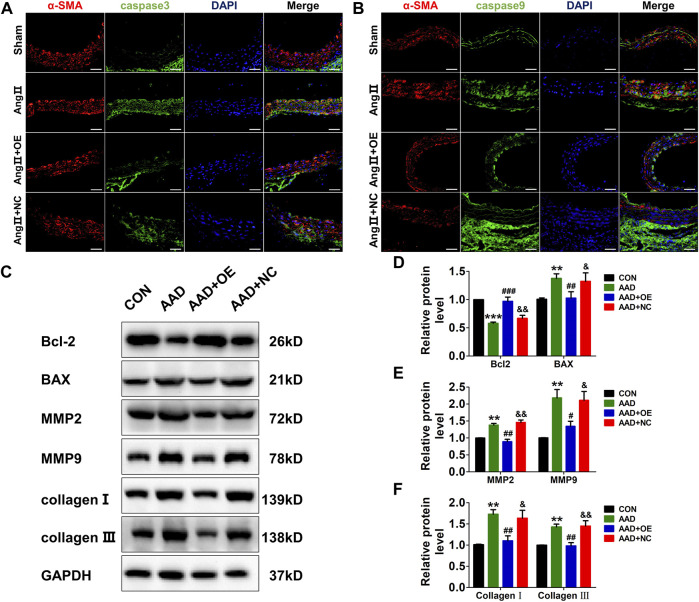
Overexpression of lnc-C2orf63-4-1 inhibits Ang II-induced vascular apoptosis, fibrosis, and MMP-2/9 activity in ApoE^−/−^ mice. **(A)** Immunofluorescent staining of α-SMA (red) and caspase-3 (green) of the aorta in all groups. (*n* = 4/per group) **(B)** Immunofluorescent staining of α-SMA (red) and caspase-9 (green) of the aorta in all groups. (*n* = 4/per group) **(C–F)** Representative Western blotting images and quantification of Bcl-2, Bax, MMP-2, MMP-9, type I collagen, and type III collagen in aorta tissue of all groups (*n* = 6/per group). GAPDH was used for internal normalization. Data of **(D–F)** were compared by analysis-of-variance (ANOVA) with Bonferroni’s post-test. **p < 0.01, ***p < 0.001 vs. control group, ^#^ *p* < 0.05, ^##^ *p* < 0.01, ^###^ *p* < 0.001 vs. AAD group, ^&^ *p* < 0.05, ^&&^ *p* < 0.01 vs. AAD + OE group. Data were all expressed as mean ± SEM.

Next, we also evaluated the expressions of apoptosis and synthetic phenotype-related markers in mouse aorta. As expected, the expressions of two apoptosis-related markers, caspase-3 and caspase-9, were dramatically increased in the AAD group, while this effect could be abolished by overexpression of lnc-C2orf63-4-1 (Figures 6A,B). Consistently, Ang II infusion significantly up-regulated pro-apoptotic protein Bax, down-regulated anti-apoptotic protein Bcl-2, elevated the expressions of MMP-2 and MMP-9, and enhanced the expressions of type I and III collagens in the mouse aorta (Figures 6C–F). After AAV9-lnc-C2orf63-4-1 treatment, the activities of these apoptosis and synthetic phenotype-related markers were attenuated (Figures 6C–F). Taken together, these results indicated that lnc-C2orf63-4-1 could abolish Ang II-elicited vascular remodeling by inhibiting the apoptosis and switched phenotype in VSMCs, which maintained the VSMC homeostasis during AAD.

### Lnc-C2orf63-4-1 Attenuates Ang II-Induced VSMC Apoptosis via STAT3 Pathways

**FIGURE 7:**
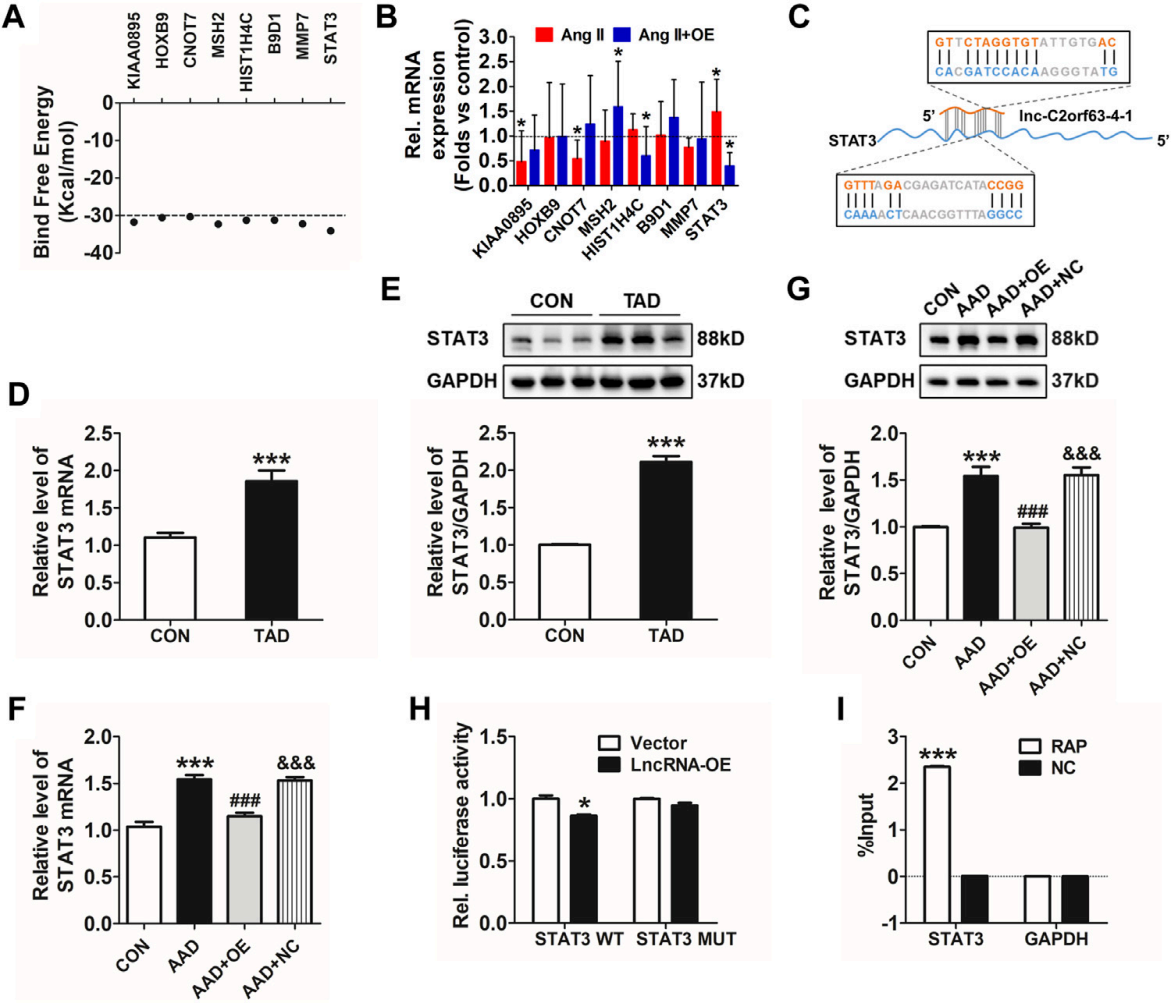
Lnc-C2orf63-4-1 directly interacts with STAT3. **(A)** Predicted panel of transcription factors potentially interacting with lnc-C2orf63-4-1, exploiting binding free energy below −30 kcal/mol. **(B)** qRT-PCR measurement of the panel of transcription factors upon AngII with or without lnc-C2orf63-4-1 (AngII + OE). **(C)** The binding sites of the STAT3 transcription factor with lnc-C2orf63-4-1 were predicted by LncTar (http://www.cuilab.cn/lnctar). **(D)** Detection of STAT3 mRNA using qRT-PCR in the aorta samples of healthy donors and clinical TAD patients (*n* = 6–10/per group). **(E)** Representative Western blotting images and quantification of STAT3 in aorta samples of healthy donors and clinical TAD patients (*n* = 6–10/per group). GAPDH was used for internal normalization. **(F)** The expression level of STAT3 in aorta tissue of all groups was evaluated by qRT-PCR (*n* = 4–6/per group). β-actin was used for normalization. **(G)** Representative Western blotting images and quantification of STAT3 in aorta tissue of all groups (*n* = 4–6/per group). GAPDH was used for internal normalization. **(H)** Dual-luciferase reporter assay of WT and Mut STAT3 3′UTR. (*n* = 4/per group). *p < 0.05 vs. Vector group. **(I)** Identification of mRNA by RAP. No RAP probes were used for the input control. Percentage of purified mRNA relative to the input group, as detected by qRT-PCR. (*n* = 3/per group). ***p < 0.001 vs. Normal group. Data of **(D,E)** were compared by Student’s t-test. Data of **(G–I)** were compared by analysis-of-variance (ANOVA) with Bonferroni’s post-test. Data were all expressed as mean ± SEM. *p < 0.05, ***p < 0.001 vs. control or vector group, ^###^ *p* < 0.001 vs. AAD group, ^&&&^ *p* < 0.001 vs. AAD + OE group.

Transcription factors are master regulators of gene expression at the transcriptional level, which control the activities of these factors to alter the transcriptome, leading to changes in cell homeostasis in response to stress. Therefore, we hypothesized that lnc-C2orf63-4-1 might interact with positive regulators of VSMC apoptosis to abrogate their homeostasis. To explore how lnc-C2orf63-4-1 regulated VSMC apoptosis, LncTar (http://www.cuilab.cn/lnctar) database was used to predict the RNA targets of lncRNAs. The analysis concluded that KIAA0895, homeobox genes B9 (HOXB9), CCR4-NOT transcription complex subunit 7 (CNOT7), histone cluster 1 H4A family member C (HIST1H4C), MMP-7, MutS Homolog 2 (MSH2), B9 protein domain 1 (B9D1), and STAT3 were possibly involved in the molecular function of lnc-C2orf63-4-1 (Figure 7A). To determine whether these mRNAs regulated the lnc-C2orf63-4-1 level as part of a feed-forward loop, we investigated their expression changes in VSMC upon overexpression of lnc-C2orf63-4-1 with Ang II stimulation. Results revealed that STAT3 was the only differentially regulated factor (Figure 7B). Bioinformatic analysis based on LncTar revealed the duplex sequence of a STAT3 binding site on lnc-C2orf63-4-1 (Figure 7C). Aberrantly increased STAT3 is involved in the vascular remodeling through promoting apoptosis (Wang et al., 2020; Wu et al., 2020). Here, we confirmed that the expression of STAT3 was significantly increased by Ang II stimulation in VSMCs, while it was restored by lnc-C2orf63-4-1 treatment (Supplementary Figures S3A,B). Consistent with a previous study, we found that the expression of STAT3 at both the mRNA and protein levels in aortic tissue was significantly increased in TAD patients compared with the healthy donors (Figures 7D,E). Meanwhile, the expression of STAT3 at the mRNA and protein levels was also elevated in the aorta of AAD mice, while it was restored by overexpression of lnc-C2orf63-4-1 (Figures 7F,G). Moreover, the efficiency of overexpression and silencing of genetic tools was also confirmed, and lnc-C2orf63-4-1 could negatively regulate the expression of STAT3 at the transcriptional level (Supplementary Figures S3C–E). These data suggested that there was a causal relationship between STAT3 and lnc-C2orf63-4-1, and the latter might act as an upstream factor of STAT3 in the development of AAD.

To further confirm the effect of lnc-C2orf63-4-1 on STAT3 in VSMCs, we performed a dual-luciferase reporter assay using a vector carrying wild-type (WT) or mutant (Mut) STAT3 3′-UTR and co-transfected them with lnc-C2orf63-4-1 mimics or NC. Luciferase reporter assays showed that lnc-C2orf63-4-1 significantly suppressed the luciferase activities in VSMCs transfected with the STAT3 3ʹ-UTR WT reporter, whereas they did not have any significant influence on the luciferase activities of the mutant reporter (Figure 7H **)**, indicating that lnc-C2orf63-4-1could directly bind to STAT3. In addition, using an RAP assay-based approach, we further confirmed that the bioinformatic analysis predicted lnc-C2orf63-4-1 binding mRNA, the STAT3, was indeed bound by lnc-C2orf63-4-1 in VSMCs (Figure 7I **)**, thus reinforcing the idea that lnc-C2orf63-4-1 maintained the VSMC homeostasis by negatively regulating STAT3.

### Overexpression of Lnc-C2orf63-4-1 Reverses VSMC Apoptosis Through Regulating Target STAT3 *in vitro*

**FIGURE 8:**
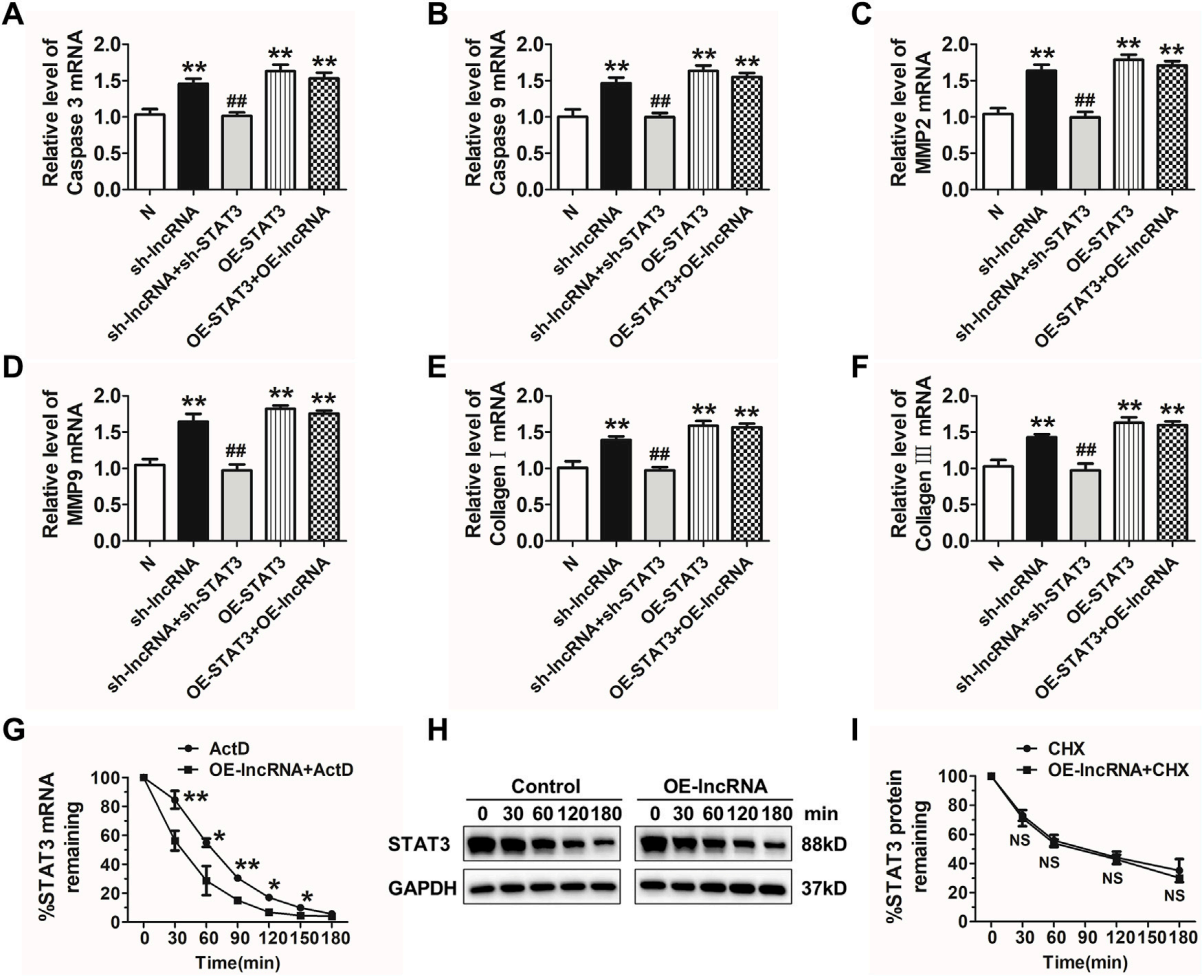
Lnc-C2orf63-4-1 restores apoptosis, fibrosis, and MMP-2/9 activity via destabilizing STAT3 mRNA in VSMCs. **(A,B)** qRT-PCR analysis results showed that lnc-C2orf63-4-1 silencing by lentivirus vector carrying a lnc-C2orf63-4-1-shRNA fragment promoted the expression of apoptosis-related markers via induction of the STAT3 (*n* = 6/per group). **(C,D)** qRT-PCR analysis results showed that lnc-C2orf63-4-1 silencing by lentivirus vector carrying a lnc-C2orf63-4-1-shRNA fragment promoted the expression of MMP-2 and MMP-9 via induction of the STAT3 (*n* = 6/per group). **(E,F)** qRT-PCR analysis results showed that lnc-C2orf63-4-1 silencing by lentivirus vector carrying a lnc-C2orf63-4-1-shRNA fragment promoted the expression of fibrosis-related markers via induction of the STAT3 (*n* = 6/per group). **(G)** Half-life (t_1/2_) of STAT3 mRNA in lnc-C2orf63-4-1 treatment by lentivirus vector carrying the lnc-C2orf63-4-1 sequence in mouse VSMCs. VSMCs were pre-incubated with actinomycin D (ActD, 5 μg/ml), then treated with lnc-C2orf63-4-1 for different time intervals (n = 6/per group). The data were presented as means ± SEM from four independent experiments in duplicate. *p < 0.05, **p < 0.01, ActD + lnc-C2orf63-4-1 vs. ActD. **(H,I)** Representative Western blotting images and relative quantification of STAT3 protein expression in mouse VSMCs. Serum-starved mouse VSMCs were pre-incubated with cyclohexamide (10 μM) for 30 min and then treated with lnc-C2orf63-4-1 by lentivirus vector carrying the lnc-C2orf63-4-1 sequence for various periods. The expression level of STAT3 at 0 min was set at 100% (*n* = 4/per group). Two-way ANOVA, lnc-C2orf63-4-1 vs. control at the same time points, NS, no significance.

Then, we explored whether deletion of STAT3 via siRNA could reverse lnc-C2orf63-4-1-induced apoptosis in VSMCs. As expected, inhibition of STAT3 reversed apoptosis-related markers and regulators of structural ECM degradation induced by silencing of lnc-C2orf63-4-1 in VSMCs (Figures 8A–F). However, overexpression of lnc-C2orf63-4-1 could not improve dysregulation of caspase-3, caspase-9, MMP-2, MMP-9, collagen I, and collagen III, which was caused by STAT3 overexpression (Figures 8A–F). These data indicated that STAT3 served as a downstream activator in the absence of lnc-C2orf63-4-1 to promote VSMC dysfunction.

Therefore, we applied actinomycin D (ActD) to test whether STAT3was post-transcriptionally regulated by lnc-C2orf63-4-1 in VSMCs. Intriguingly, the half-life of STAT3 mRNA was markedly reduced in response to overexpression of lnc-C2orf63-4-1 (Figure 8G), indicating that lnc-C2orf63-4-1 might regulate the expression of STAT3 via mRNA destabilization. Moreover, we applied cycloheximide (CHX) to inhibit *de novo* protein synthesis. As shown in Figures 8H,I, the half-life of STAT3 protein was not affected by CHX. Taken together, these results suggested that enhanced stabilization of STAT3 mRNA in VSMCs increased the expression of STAT3, which might be attributed to lnc-C2orf63-4-1 deficiency during Ang II stimulation.

## Discussion

Acute AAD is a severe and life-threatening cardiovascular disease without effective pharmacotherapy at present. Hence, it is necessary to investigate the biological basis of aortic dissection and identify novel targets for prevention and therapy. In the present study, we identified an uncharacterized lncRNA, lnc-C2orf63-4-1, which was down-regulated in the aorta of TAD patients and functionally required for the vascular remodeling. We generated mice non-specifically overexpressing lnc-C2orf63-4-1 using the AAV-9 vector and showed that up-regulation of lnc-C2orf63-4-1 greatly attenuated Ang II-induced AAD. Following Ang II infusion, the ApoE^−/−^ mice exhibited more severe apoptosis, greater MMP-2/9 activity, less contractile phenotype of SMC, and increased collagen deposition compared with the control mice. Mechanistic studies revealed that Ang II infusion resulted in a marked increase in the expressions of apoptosis-related gene caspase-3/9 and Bax, elevated expressions of interstitial fibrosis-related genes collagen type I/III, and enhanced STAT3 transcription activity, which was partially restored by pre-treatment of lnc-C2orf63-4-1. Moreover, STAT3 was identified as a downstream target of lnc-C2orf63-4-1 through bioinformatics analysis, and lnc-C2orf63-4-1 also attenuated the increased expression of STAT3 mediated apoptosis and synthetic phenotypic transformation in VSMCs induced by Ang II stimulation. Collectively, our findings demonstrated that lnc-C2orf63-4-1 played an important role in regulating vascular remodeling and pathogenesis of aortic dissection. Recently, new research has been proposed that Ang II-mediated up-regulation of lncRNA lnc-OIP5-AS1 results in increased expressions of apoptotic genes as a competing endogenous RNA of microRNAs in human aortic VSMCs and exacerbates aortic injury (Wang et al., 2021), and a new mechanism has demonstrated that lncRNA GAS5 in AAD promotes VSMC apoptosis and represses its proliferation by inducing YBX1 to regulate the downstream target p21 (He et al., 2019). From these studies, a broader understanding of the mechanisms underlying the action of more lncRNAs might further facilitate the development of new therapeutic strategies for AAD.

LncRNAs are defined as transcripts longer than 200 nt without evident protein-coding function, and they are located in both the nucleus and cytoplasm and participate in the regulation of gene expression at both the transcriptional and post-transcriptional levels to affect numerous physiological and pathological processes (Batista and Chang, 2013; Engreitz et al., 2016; Li et al., 2018). For example, Li and Yang (2018) have identified 1,352 up-regulated and 1,624 down-regulated lncRNAs between thoracic aortic aneurysm and normal thoracic aorta by using lncRNA microarray, and the bioinformatics analysis has revealed that an lnc-RP11-465L10.10 may participate in regulating the transcription of MMP-9, however, the number of samples is not expanded to confirm the accuracy of microarray that would limit the data reliability. Recently, Li et al. (2018b) have demonstrated the differentially expressed lncRNAs between TAD and normal thoracic aorta, while the number of samples to validate the microarray by qRT-PCR is small, and the potential function of target lncRNA is not revealed by *in vivo* or *in vitro* study. These studies indicate that the differentially expressed lncRNAs may play an important role in the development of AAD, and it is urgently necessary to understand the potential mechanisms and pathways of target lncRNAs to affect the function of VSMCs. In the present study, we identified 53 lncRNAs that were aberrantly expressed between healthy donors and TAD patients by using HTS analysis. The accuracy of transcriptome sequencing was confirmed by a relative large number of samples. Moreover, we found that the lnc-C2orf63-4-1 was a lowly expressed novel lncRNA in the aorta of TAD patients, which was significantly correlated with increased aortic arch width, suggesting that lnc-C2orf63-4-1 played a critical role in vascular remodeling during AAD.

AAD is characterized by extensive molecular changes in vascular remodeling and loss of the contractile phenotype of VSMCs. Multiple mechanisms have been proposed to promote the pathogenesis of AAD, including VSMC apoptosis, ECM degradation, and vascular interstitial fibrosis. As the predominant cells in the AAD of the aorta, VSMCs are essential for the maintenance of the aortic structure and function. Apoptosis of VSMCs would reduce the tensile strength and elasticity of the aorta, resulting in tunica media susceptible to rupture and contributing to the development and progression of AAD (Henderson et al., 1999; Ailawadi et al., 2009). Herein, we found that the apoptosis-related markers were remarkedly up-regulated in VSMCs and ApoE^−/−^mice by Ang II stimulation compared with the control group. Moreover, the AAD mice showed enhanced vascular interstitial fibrosis, which was mainly attributed to the activation of collagen type I and III proteins in VSMCs. In addition, changes in MMPs are another important mechanism for the development of AAD. When VSMCs switch from a contractile phenotype to a synthetic phenotype under pathological conditions, they promote a proinflammatory response and increase the production of MMPs. Both aorta samples from TAD patients and AAD animals exhibited an enhanced production and secretion of MMPs, especially MMP-2 and MMP-9, leading to augmented elastin degradation and aortic wall weakening, and ultimately rendering the aorta prone to rupture and AAD progression (Fan et al., 2019; Xu et al., 2019). Herein, the AAD mice also showed markedly increased vascular ECM degradation by EVG staining, which could be attributed to the induced expressions of MMP-2/9 in Ang II-induced VSMC apoptosis. More importantly, these above pathologic changes of vascular remodeling in AAD mice or Ang II-induced VSMCs were partly attenuated by lnc-C2orf63-4-1 treatment.

LncRNAs have been reported to be located in both the nucleus and the cytoplasm, and subcellular localization patterns of lncRNAs reveal fundamental insights into their biology and provide hypotheses for potential molecular roles (Derrien et al., 2012; Fort et al., 2014). In the present study, we found that lnc-C2orf63-4-1 was localized in the cytoplasm of VSMCs when we performed an RNA-FISH assay of aorta samples, indicating that lnc-C2orf63-4-1 might participate in the post-transcriptional regulation process. Recently, a novel mechanism of post-transcriptional regulation has shown that lncRNAs function as a molecular scaffold or decoy, interfere with mRNA pathways, and guide proteins to specific genetic loci by mediating RNA-RNA interaction (Gu et al., 2019). Given the cellular effects of lnc-C2orf63-4-1 modulation in fate decisions of VSMCs, we speculated that lnc-C2orf63-4-1 could be operated through transcription factors in AAD progression as well. Therefore, we selected a panel of transcription factors with predicted binding to lnc-C2orf63-4-1. STAT3 was the only dysregulated transcription factor in response to overexpression and Ang II stimulation in VSMCs. Functional experiments indicated that STAT3 was essential in mediating the anti-apoptotic effects of lnc-C2orf63-4-1. Interestingly, an increased level of STAT3 has been identified in abdominal aortic aneurysms by Cai et al., showing that STAT3 acts as a transcription factor and contributes to lncRNA-NEAT1 transcription to facilitate aortic aneurysm formation (Cai et al., 2020), which is also consistent with our results. As lncRNA could regulate the expression of STAT3 at the transcriptional or post-transcription level, we next applied transcription inhibitor and protein synthesis inhibitor to test whether STAT3 was post-transcriptionally regulated by inhibiting *de novo* synthesis of mRNA or protein in VSMCs, respectively. Intriguingly, the half-life of STAT3 mRNA was markedly reduced in response to lnc-C2orf63-4-1, while the level of STAT3 protein was not affected by lnc-C2orf63-4-1, indicating that lnc-C2orf63-4-1 regulated the STAT3 expression via destabilizing mRNA.

It has been reported that Ang II can elicit phosphorylation and activation of STAT3, and then STAT3 forms homodimers or heterodimers translocated to the nucleus and activates the transcription of target genes (Murray, 2007). Tissues from AAD patients express high levels of STAT3 and exhibit apoptosis in VSMCs of the tunica media (Ju et al., 2013). A more recent study has found that Ang II-induced AAD formation in ApoE^−/−^ mice depends at least in part on the Ang II-mediated activation of STAT3 (Ohno et al., 2018). Moreover, STAT3 inhibitor has been shown to significantly inhibit the formation of AAD in an animal model (Wu et al., 2020). Upon exposure to the pathological stimulus, VSMCs show enhanced expressions of apoptosis-related genes, including caspase family and Bcl-2 family, contributing to the loss of VSMCs that led to aortic wall weakening and rupture (Luo et al., 2020). Indeed, STAT3 acts as the upstream introducer of apoptosis in many types of cells, and the STAT3 inhibitor has been widely used in the field of anti-tumor treatment via suppressing apoptosis. Consistent with the previous studies, we found that the lnc-C2orf63-4-1 could attenuate the apoptosis of VSMCs induced by Ang II stimulation, while overexpression of STAT3 would counteract the protective effect of lnc-C2orf63-4-1, suggesting that STAT3 acted as a downstream factor of lnc-C2orf63-4-1 in regulating VSMC apoptosis. More importantly, the lnc-C2orf63-4-1 and the STAT3 3ʹUTR have been shown to interact in VSMCs by RAP assays. Co-transfection of lnc-C2orf63-4-1 and pGL3 constructs containing a STAT3 3ʹUTR sequence inhibited the luciferase activity, supporting the idea that lnc-C2orf63-4-1 directly inhibited the expression of STAT3.

Our study also has some limitations. First, we only detected the expression of lnc-C2orf63-4-1 from aorta samples but not in the blood samples of TAD patients. Multiple confirmations would enhance the evidence of lnc-C2orf63-4-1 as a clinical biomarker in AAD. Second, we only identified that lnc-C2orf63-4-1 could negatively regulate the expression of STAT3 at the transcriptional level through stabilizing STAT3 mRNA in VSMCs. Whether lnc-C2orf63-4-1 could function as a sponge lncRNA to affect the expressions of other key regulators in AAD remains largely unexplored. Third, we did not provide evidence of whether the lnc-C2orf63-4-1-transgenic mice could prevent Ang II-induced AAD. Nonetheless, the present findings in human aortic dissection samples and AAD mouse model generated novel hypotheses about the role of lnc-C2orf63-4-1 in the progression of AAD.

## Data Availability

The datasets presented in this study can be found in online repositories. The names of the repository/repositories and accession number(s) can be found below: Sequence Read Archive (SRA) of RNA-seq data, https://submit.ncbi.nlm.nih.gov/subs/sra/SUB10522213/overview. Accession number: SRR16362695, SRR16362694, SRR16362693, SRR16362692, SRR16362691, SRR16362690.
