# Supplementary material for: Lnc-C2orf63-4-1 Confers VSMC Homeostasis and Prevents Aortic Dissection Formation via STAT3 Interaction
Source: Front Cell Dev Biol. 2021 Dec 6;9:792051. doi: 10.3389/fcell.2021.792051 (PMC8685433; doi:10.3389/fcell.2021.792051)

Title: Lnc-C2orf63-4-1 Confers VSMC Homeostasis and Prevents Aortic Dissection Formation via STAT3 Interaction

Running title: lnc-C2orf63-4-1 in aortic dissection disease

Song Zhang, PhD [1*]; Shiqi Zhao, PhD [1*]; Xuejie Han, PhD [1*]; Yun Zhang, PhD [1]; Xuexin Jin, PhD [7]; Yue Yuan, PhD [1]; Xinbo Zhao, PhD [1]; Yingchun Luo, PhD [1]; Yun Zhou, MS[1]; Yunlong Gao, MS[1]; Hui Yu, MS[1]; Danghui Sun, PhD [1], Wei Xu, PhD [1]; Sen Yan, PhD [1]; Yongtai Gong, MD, PhD [1#]; Yue Li, MD, PhD [1,2,3,4,5,6#]

[1]Department of Cardiology, the First Affiliated Hospital, Harbin Medical University, Harbin 150001, China; [2]The Cell Transplantation Key Laboratory of National Health Commission, Harbin 150001, China; [3]Key Laboratory of Hepatosplenic Surgery, Harbin Medical University, Ministry of Education, Harbin 150001, China; [4]Key Laboratory of Cardiac Diseases and Heart Failure, Harbin Medical University, Harbin 150001, China; [5]Institute of Metabolic Disease, Heilongjiang Academy of Medical Science, Harbin 150081, China; [6]Heilongjiang Key Laboratory for Metabolic Disorder & Cancer Related Cardiovascular Diseases, Harbin 150001, China; [7]Department of Pharmacology (State-Province Key Laboratories of Biomedicine-Pharmaceutics of China, Key Laboratory of Cardiovascular Medicine Research, Ministry of Education), College of Pharmacy, Harbin Medical University, Harbin, China.

*These authors contribute equally to this paper.

#Address for correspondence:

Yue Li, Department of Cardiology, the First Affiliated Hospital, Harbin Medical University, Youzheng Street No. 23, Nangang District, Harbin 150001, China.

Tel: +86 451 85555673; Fax: +86 451 53675733

E-mail addresses: ly99ly@hrbmu.edu.cn

Supplemental Table 1. Clinical characteristics of TAD patients and Control subjects.

	TAD patients （n=24）	Control subjects （n=13）	*P* value
Age（years）	52.5±10.9	57±16.1	0.0938
Male（%）	17(70.8)	10(76.9)	
Hypertension	11(45.8)	5(38.4)	
Smoking	15(62.5)	6(46.2)	
Ascending aorta diameter （mm）	40.8±7.21	30.8±3.42	＜0.001

Values are n (%) or mean ± standard deviation. $P < 0.05$ was considered statistically significant. TAD indicates thoracic aortic dissection.

Supplemental Table 2. Primer Sequence.

Gene	F or R	Primer Sequence
KIAA0895	F	TCGCGTAACGGTCCATCAAT
	R	CTGGAAAGCAGGTCTTCGGT
HOXB9	F	CCCAAGTGAGTGGGGAAGAG
	R	AGCGAGGCTCATCACTTTTCT
CNOT7	F	ATGTACAGAACGGCACAGGG
	R	ACTGAAAGCAGAGAAGGTCTACA
HIST1H4C	F	TGAAGGTGTTCCTGGAGAACG
	R	GCCTTTTGTTGACAGTGGAAATC
MMP7	F	TAGTTGGGGGACTGCGGATA
	R	CAGGAAGTTCACTCCTGCGT
MSH2	F	ATTGGAACCTCGCTGGGATG
	R	GATGCTCTCCTCCGACATGG
B9D1	F	TGACTCGTGTTCGCTCTCAG
	R	GCGGACAGAAGGGCTGATTT
STAT3	F	GCCACGTTGGTGTTTCATAATC
	R	TTCGAAGGTTGTGCTGATAGAG
Caspase3	F	AGATGGCTTGCCAGAAGATAC
	R	CTGCAAAGGGACTGGATGAA
Caspase9	F	GTGACATCCTTGTGTCCTACTC
	R	CAGCCAGGAATCTGCTTGTA
MMP2	F	CTGGAATGCCATCCCTGATAA
	R	GGTTCTCCAGCTTCAGGTAATAA
MMP9	F	GTGGAACTCACACGACATCTT
	R	TCCACCTTGTTCACCTCATTT
COL1A1	F	AGACCTGTGTGTTCCCTACT
	R	GAATCCATCGGTCATGCTCTC
COL3A1	F	GTGACTCAGGATCTGTCCTTTG

	R	GTAGAAGGCTGTGGGCATATT
β-actin	F	GGACCTGACAGACTACCTCAT
	R	GCTCGAAGTCTAGAGCAACATAG

Supplemental Table 3. General information of top 10 ranked up- and downregulated lncRNAs.

LncRNA ID	*P* value	Fold change	Up or Down	Locus
ENST00000624848	7.01E-05	8.33	Up	10:48443836-48445820
NONHSAT199898.1	5.95E-05	7.74	Up	4:40542684-40630809
NONHSAT172866.1	0.000118639	7.49	Up	16:30722160-30724450
NONHSAT001553.2	6.28E-05	7.47	Up	1:23771130-23778385
NONHSAT162346.1	1.42E-05	7.14	Up	12:65806349-65853393
ENST00000475947	8.06E-06	7.09	Up	17:16438986-16441733
NONHSAT223202.1	8.37E-05	6.86	Up	X:13889987-13898741
NONHSAT199001.1	9.86E-05	6.76	Up	4:119212648-119259919
NONHSAT052793.2	0.000112907	6.53	Up	17:30600375-30633491
NONHSAT120405.2	1.28E-07	6.24	Up	7:44876301-44878985
MSTRG.16970.1	2.33E-07	-9.61	Down	10:47115477-47118785
NONHSAT223595.1	3.06E-07	-8.85	Down	X:136872625-136872845

ENST00000620266	1.40E-06	-8.64	Down	17:68126666-68129586
NONHSAT179882.1	1.54E-06	-7.97	Down	19:49684789-49688445
ENST00000414046	2.30E-06	-7.63	Down	6:31400702-31465809
ENST00000415675	2.65E-06	-7.17	Down	1:159194325-159202512
NONHSAT220386.1	3.85E-06	-7.12	Down	9:136670711-136672675
NONHSAT106677.2	5.33E-06	-6.94	Down	6:2452411-2484027
ENST00000622750	9.24E-06	-6.80	Down	17:68133201-68135935
NONHSAT070752.2	5.66E-05	-6.24	Down	2:54972559-54972776

Supplemental Figures and Figure Legends

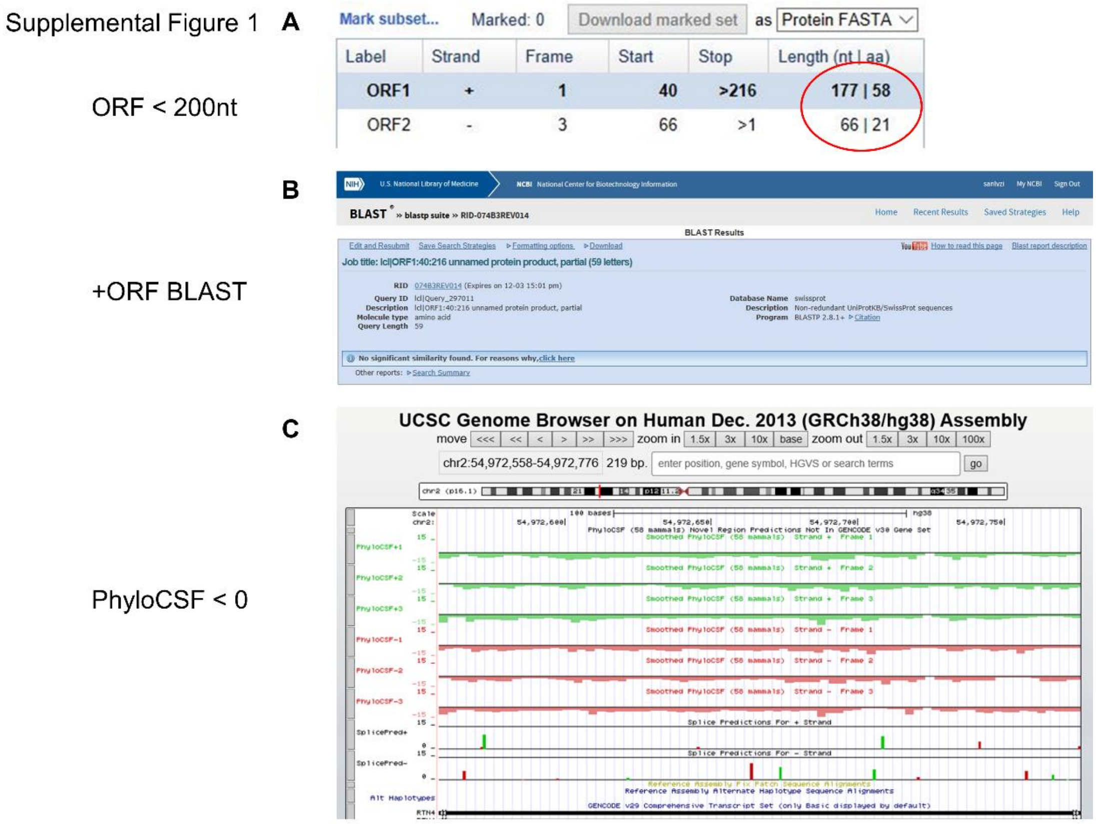

Figure S1. Identification of lnc-C2orf63-4-1 and its non-coding nature.

(A-B) Prediction of putative proteins encoded by lnc-C2orf63-4-1 using ORF Finder.

(C) The codon substitution frequency scores (CSF) of lnc-C2orf63-4-1.

Supplemental Figure 2

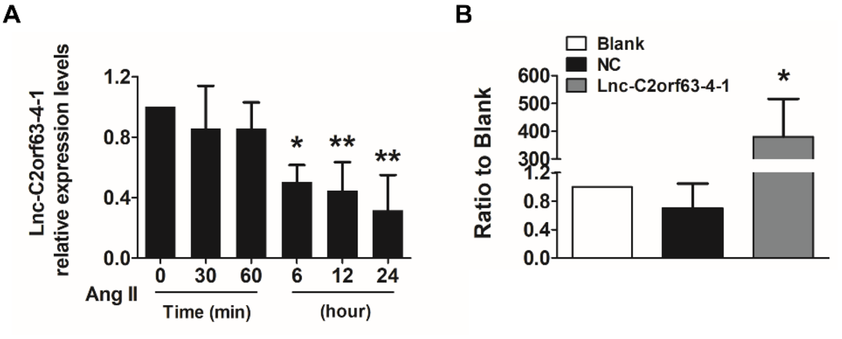

Figure S2. AngII inhibited the expression of lnc-C2orf63-4-1 *in vitro*.

(A) qRT-PCR analysis of lnc-C2orf63-4-1. Mouse VSMCs were treated with Ang-II at the indicated time, and the expression of lnc-C2orf63-4-1 was analyzed (n = 6/per group). **(B)** The effects of adenovirus vector carrying overexpression of lnc-C2orf63-4-1 treatment in mouse VSMCs by qRT-PCR (n = 6/per group). Data are all compared by analysis-of-variance (ANOVA) with Bonferroni's post-test. **P* < 0.05, ***P* < 0.01 vs control or blank group. Data are all expressed as mean±SEM.

Supplemental Figure 3

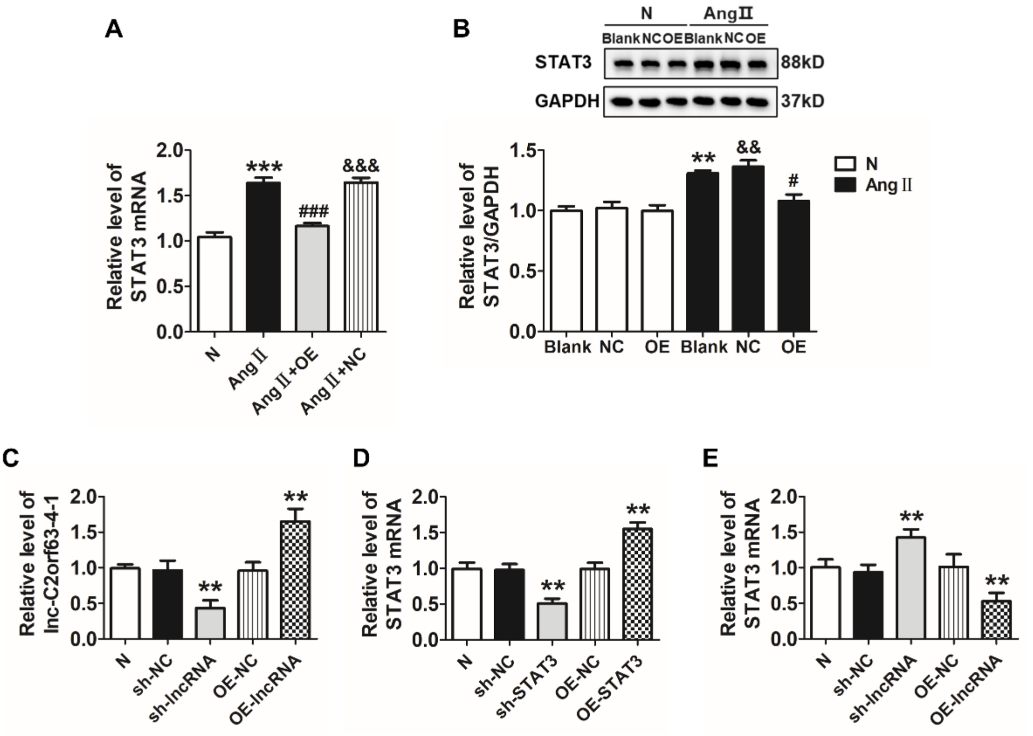

Figure S3. Knockdown of lnc-C2orf63-4-1 increased STAT3 levels by lnc-C2orf63-4-1 shRNA *in vitro*.

(A) The mRNA levels of STAT3 were upregulated on Ang-II treatment, while it was reversed by overexpression of lnc-C2orf63-4-1 in mouse VSMCs (n = 6/per group). ****P*<0.001 vs N group, ###*P*<0.001 vs AngII group, &&*P*<0.01 vs AngII+OE group. N, normal group represents VSMCs treatment with cell culture medium. Blank, VSMCs treatment with empty vector. **(B)** The protein levels of STAT3 were upregulated on Ang-II treatment, while it was reversed by overexpression of lnc-C2orf63-4-1 in mouse VSMCs (n = 6/per group). ***P*<0.01 vs N or N+Blank group, &&*P*<0.01 vs AngII+OE group. #*P*<0.05 vs AngII+Blank group. **(C)** The effects of shRNA or overexpression vector of lnc-C2orf63-4-1 in mouse VSMCs by qRT-PCR (n = 6/per

group). **(D)** The effects of shRNA or overexpression vector of STAT3 in mouse VSMCs by qRT-PCR (n = 6/per group). **(E)** The effect of lncRNA on STAT3 expression in mouse VSMCs by qRT-PCR (n = 6/per group). $^{**}P<0.01$ vs sh-NC group. sh-NC, VSMCs treatment with short hairpin RNA vector carrying negative control gene. Data are all compared by analysis-of-variance (ANOVA) with Bonferroni's post-test. Data are all expressed as mean±SEM.

Original picture of western blot

Figure 3A

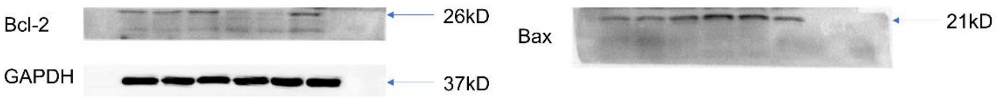

Figure 3F

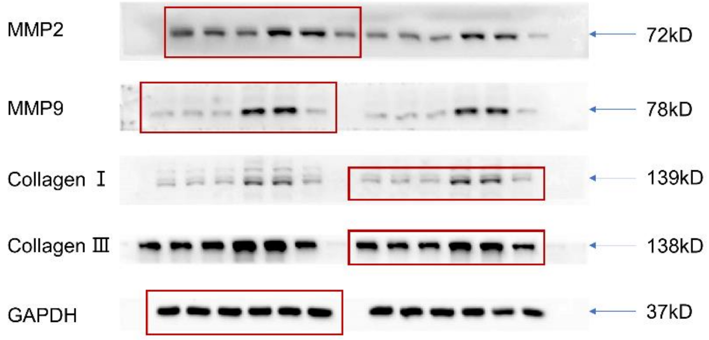

Figure 6C

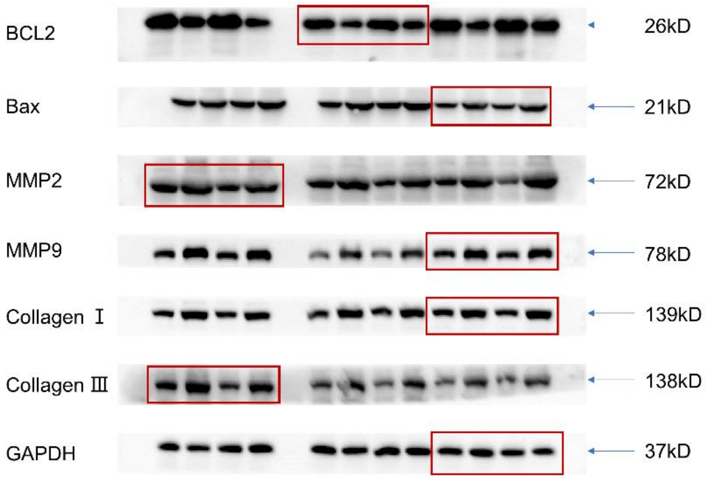

Figure 7E

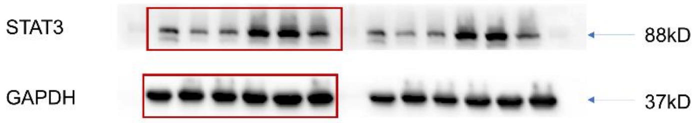

Figure 7G

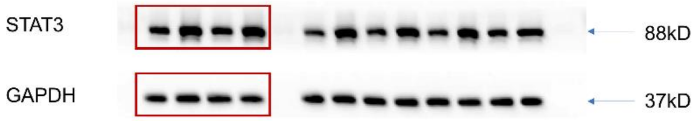

Figure 8H

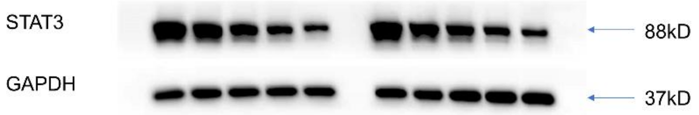

Supplemental Figure 3B

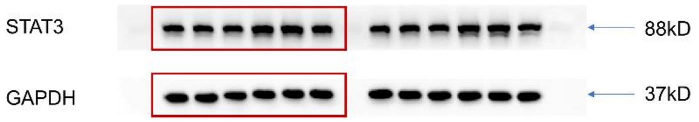

Supplement: Supplementary file 1 [file DataSheet1.PDF]
